# Intestinal Microbial Metabolic Activation Converts Soy Isoflavones into Potent Direct Antioxidants: First Total Synthesis and Mechanistic Characterization of 5-Hydroxydehydroequol

**DOI:** 10.3390/antiox15101240

**Published:** 2026-09-25

**Authors:** Sakurako Okada, Yoshimi Shoji, Masao Morita, Mika Hayashi, Wakana Shimizu, Kei Ohkubo, Hiromu Ito, Ikuo Nakanishi, Kiyoshi Fukuhara

**Affiliations:** 1 Division of Organic and Medicinal Chemistry, Showa Medical University School of Pharmacy, Shinagawa-ku, Tokyo 142-8555, Japan; ikeda-s@cmed.showa-u.ac.jp (S.O.); masao.morita@showa-u.ac.jp (M.M.); ep21m132@edu.showa-u.ac.jp (M.H.); shimizu.wkn@cmed.showa-u.ac.jp (W.S.); 2 Quantum RedOx Chemistry Team, Quantum Life Spin Group, Institute for Quantum Life Science (iQLS), National Institutes for Quantum Science and Technology (QST), Chiba-shi 263-8555, Chiba, Japan; shoji.yoshimi@qst.go.jp (Y.S.); ito.hiromu@qst.go.jp (H.I.); nakanishi.ikuo@qst.go.jp (I.N.); 3 Institute for Open and Transdisciplinary Research Initiatives (OTRI), The University of Osaka, Suita 565-0871, Osaka, Japan; ohkubo@irdd.osaka-u.ac.jp

**Keywords:** genistein, daidzein, equol, phenoxodiol, 5-hydroxydehydroequol, antioxidant activity, radiation-induced oxidative damage

## Abstract

Soy isoflavones exhibit diverse health-promoting effects, including antioxidant and anti-inflammatory activities. However, these biological effects have largely been attributed to the modulation of cellular signaling pathways, whereas the influence of intestinal microbial metabolism on their direct radical-scavenging properties remains poorly understood. In this study, synthetic routes were established for the major intestinal bacterial metabolites daidzein and genistein, equol (Eq), dehydroequol (DEq), 5-hydroxyequol (5-OH-Eq), and, for the first time, 5-hydroxydehydroequol (5-OH-DEq), and their direct radical-scavenging activities, reaction mechanisms, radical-scavenging capacities, and DNA protective effects were systematically evaluated. Introduction of a C-ring double bond markedly enhanced direct radical-scavenging activity, whereas the parent isoflavones and their reduced metabolites exhibited little or no activity. Among the compounds examined, 5-OH-DEq exhibited the highest antioxidant activity, scavenging radicals predominantly through hydrogen atom transfer, with a second-order rate constant of 1.44 × 10^3^ M^−1^ s^−1^, exceeding that of Trolox and a radical-scavenging capacity of approximately three radical equivalents per molecule. Moreover, 5-OH-DEq protected plasmid DNA against radiation-induced oxidative damage. These findings demonstrate that intestinal microbial metabolism can function as a form of metabolic activation, converting dietary isoflavones into potent direct antioxidants. 5-OH-DEq was identified as a previously unexplored antioxidant metabolite and a promising lead scaffold for further development of next-generation phenolic antioxidants.

## 1. Introduction

Phytoestrogens are naturally occurring polyphenolic compounds that structurally resemble endogenous estrogens and exhibit mild estrogenic activity in humans [1]. Soy isoflavones, such as daidzein and genistein, have attracted considerable attention because of their diverse biological activities, including antioxidant, anti-inflammatory, cardioprotective, and anticancer effects [2,3,4,5,6,7,8]. Epidemiological and clinical studies have associated dietary soy isoflavone intake with reduced risks of cardiovascular disease, osteoporosis, nonalcoholic fatty liver disease, and hormone-dependent cancers, particularly breast cancer [5,9,10,11]. These effects have generally been attributed to estrogen receptor modulation and regulation of oxidative stress and inflammatory signaling pathways [3,6,7,12,13].

However, accumulating evidence suggests that the biological activities of soy isoflavones depend not only on the parent compounds but also on metabolites generated by the intestinal microbiota [14,15,16,17]. Following oral administration, daidzein and genistein undergo extensive microbial biotransformation in the colon, producing metabolites with biological properties distinct from those of their parent isoflavones [15,18,19,20]. Among these metabolites, equol (Eq), produced from daidzein, has been extensively investigated because of its high estrogen receptor affinity and greater biological activity [12,13,17,20,21]. More recently, dehydroequol (DEq), an unsaturated metabolite containing a C-ring double bond, was identified in microbial transformation systems (Figure 1) [14,15]. DEq exhibits greater antiproliferative activity than Eq and has attracted considerable interest as the core structure of the anticancer drug candidate phenoxodiol [22,23,24,25]. These findings indicate that intestinal microbial metabolism can markedly alter the pharmacological properties of dietary isoflavones.

Genistein undergoes an analogous metabolic pathway to produce 5-hydroxyequol (5-OH-Eq) and the corresponding unsaturated metabolite, 5-hydroxydehydroequol (5-OH-DEq) [14,15,17]. However, despite their potential biological importance, little is known about the physicochemical properties or biological activities of these metabolites [14,15]. Several chemical syntheses of Eq and DEq have previously been reported. For example, Gupta and Ray constructed a protected chromanone framework from substituted phenols and phenylacetic acid derivatives, followed by reduction, dehydration, and high-temperature deprotection to obtain Eq and DEq [26]. Wähälä and co-workers also reported the synthesis of tetrahydrodaidzein-related intermediates through stepwise reduction in the isoflavone framework [27]. Although these studies established chemical access to daidzein-derived metabolites, the reported procedures generally required multiple protection, ring-construction, reduction, and deprotection steps. In contrast, to the best of our knowledge, a chemical total synthesis of 5-OH-DEq has not previously been reported. The lack of a reliable synthetic method for 5-OH-DEq has prevented ready access to this metabolite and consequently limited detailed investigation of its physicochemical properties and biological activities.

From a chemical perspective, introducing a C-ring double bond converts the isoflavan framework into a stilbene-like conjugated structure, extending π-conjugation throughout the molecule [15,28]. This structural modification is expected to stabilize the phenoxyl radical formed during hydrogen atom transfer, increase the HOMO energy level, and substantially enhance direct radical-scavenging activity [29,30,31,32]. In contrast to conventional soy isoflavones, which generally exhibit only modest direct antioxidant activity because they lack a catechol moiety [33,34], dehydrogenation may transform these metabolites into highly efficient direct antioxidants. Furthermore, the additional 5-hydroxyl group in 5-OH-DEq is expected to enhance electron donation and provide an additional hydrogen atom donor, potentially improving both the kinetics and capacity of radical scavenging.

Based on these considerations, we hypothesized that intestinal microbial metabolism represents a form of metabolic activation that converts soy isoflavones into structurally optimized antioxidants. To test this hypothesis, we developed synthetic routes to the major metabolites of daidzein and genistein, including the first total synthesis of 5-OH-DEq and an improved synthesis of DEq. We systematically investigated the radical-scavenging activity, reaction kinetics, reaction mechanisms, radical-scavenging capacity, and DNA-protective effects. Our results demonstrate that C-ring unsaturation dramatically enhances the direct antioxidant properties of isoflavone metabolites and identify 5-OH-DEq as a previously unexplored, highly potent antioxidant metabolite generated by intestinal microbial metabolism.

## 2. Materials and Methods

### 2.1. General Methods

Unless otherwise noted, all commercially available reagents and solvents were purchased from standard commercial suppliers and used without further purification. Solvents used for column chromatography were of industrial grade. The progress of all reactions was monitored by thin-layer chromatography (TLC) on silica gel 60 F254 plates (0.25 mm, Merck, Darmstadt, Germany). Preparative TLC for compound purification was performed using Merck silica gel plates (20 × 20 cm, layer thickness 2000 μm). Flash column chromatography was performed using silica gel 60 (spherical, 63–210 μm). ^1^H and ^13^C NMR spectra were recorded on a JEOL JNM-AL500 spectrometer (500 MHz, JEOL Ltd., Tokyo, Japan) using CDCl_3_, CD_3_OD, or DMSO-*d*_6_ as the solvent, with tetramethylsilane (TMS) as the internal standard. High-resolution mass spectra (HRMS) were obtained on a JMS-T100LP AccuTOF LC-Express time-of-flight mass spectrometer equipped with an atmospheric pressure ionization (API) source (JEOL Ltd., Tokyo, Japan). The purity of the final synthesized compounds was assessed by ^1^H NMR spectroscopy based on the relative intensities of the signals attributable to the target compounds and detectable impurities. The structures of the synthesized compounds were confirmed by ^1^H and ^13^C NMR spectroscopy and HRMS ^1^H NMR and ^13^C NMR spectra of the final compounds Eq, DEq, 5-OH-Eq, and 5-OH-DEq are provided in the Supporting Information.

### 2.2. Synthesis of Eq

Daidzein (1.00 g, 3.93 mmol) was placed in a stainless-steel high-pressure reactor equipped with a Teflon liner, and 10% Pd/C (1.00 g) and MeOH (10 mL) were added. The reactor was charged with H_2_ (50 atm), and the mixture was stirred at 80 °C for 24 h. After cooling to room temperature, the catalyst was removed by filtration through a Celite pad, and the filtrate was concentrated under reduced pressure. The entire resulting crude residue was divided into three approximately equal portions, each corresponding to one-third of the crude reaction mixture derived from approximately 333 mg of the starting daidzein, and each portion was applied to a separate preparative silica gel TLC plate (20 × 20 cm, layer thickness 2000 μm). The three plates were developed individually using hexane/EtOAc (1:1, *v*/*v*). The product-containing band from each plate was collected, and the silica gel was extracted with EtOAc. The extracts obtained from all three plates were combined and concentrated under reduced pressure to afford Eq as a white powder (590 mg, 2.44 mmol, 62%). ^1^H NMR (500 MHz, DMSO-d_6_) δ 9.27 (1H, br s, 7-OH), 9.15 (1H, br s, 4′-OH), 7.07 (2H, d, *J* = 8.5 Hz, H-2′,6′), 6.83 (1H, d, *J* = 8.2 Hz, H-5), 6.68 (2H, d, *J* = 8.5 Hz, H-3′,5′), 6.24 (1H, dd, *J* = 8.2, 2.7 Hz, H-6), 6.15 (1H, d, *J* = 2.7 Hz, H-8), 4.11 (1H, m, H-2), 3.87 (1H, m, H-2), 2.97 (1H, m, H-3), 2.77 (2H, m, H-4). ^13^C NMR (125 MHz, CD_3_OD) δ 156.3, 156.0, 155.0, 132.5, 129.9, 128.0, 115.1, 113.3, 107.8, 102.5, 70.9, 38.1, 31.7. HRMS (DART-MS) *m*/*z* [M + H]^+^ calcd for C_15_H_15_O_3_ 243.1021, found 243.1028.

### 2.3. Synthesis of DEq

#### 2.3.1. 7-Hydroxy-3-(4-hydroxyphenyl)chroman-4-one (**1**)

Daidzein (5.00 g, 19.7 mmol) was dissolved in MeOH (400 mL), and 10% Pd/C (2.30 g) and ammonium formate (6.10 g, 96.7 mmol) were added. The reaction mixture was heated at reflux (85 °C) for 150 min. The reaction was monitored by TLC (hexane/EtOAc = 1:2). After completion, the catalyst was removed by filtration through Celite, and the filtrate was concentrated under reduced pressure. The resulting residue was used directly in the next step without further purification.

#### 2.3.2. 3-(4-Hydroxyphenyl)chromane-4,7-diol (**2**)

The crude compound **1** obtained in the preceding step was dissolved in THF (200 mL). A solution of LiBH_4_ (4.0 M in THF, 53 mL, 208 mmol) was diluted with THF (150 mL) and cooled in an ice bath. The solution of 1 was added dropwise to the LiBH_4_ solution under stirring. After the addition was complete, the reaction mixture was stirred at room temperature for 17 h. The reaction was monitored by TLC (hexane/EtOAc = 1:1). The reaction was quenched with saturated aqueous NH_4_Cl, and the organic layer was washed with brine, dried over anhydrous Na_2_SO_4_, filtered, and concentrated under reduced pressure. The resulting residue was used directly in the next step without further purification.

#### 2.3.3. 3-(4-Hydroxyphenyl)-2H-chromen-7-ol (DEq)

The crude compound **2** obtained in the preceding step was dissolved in THF (200 mL), and trimethylsilyl trifluoromethanesulfonate (TMSOTf, 4.33 mL, 24.0 mmol) was added at −20 °C. The reaction mixture was stirred at the same temperature for 15 min. The organic layer was washed with brine, dried over anhydrous Na_2_SO_4_, filtered, and concentrated under reduced pressure. The residue was purified by silica gel column chromatography (hexane/EtOAc = 2:1) to afford DEq as a pale pink solid (981.9 mg, 4.09 mmol, 21% overall yield from daidzein). ^1^H NMR (500 MHz, DMSO-d_6_) δ 9.55 (1H, br s, OH), 7.29 (2H, d, *J* = 8.3 Hz, H-2′,6′), 6.90 (1H, d, *J* = 8.5 Hz, H-5), 6.75 (2H, d, *J* = 8.3 Hz, H-3′,5′), 6.72 (1H, s, H-4), 6.30 (1H, dd, *J* = 8.5, 2.5 Hz, H-6), 6.22 (1H, d, *J* = 2.5 Hz, H-8), 4.99 (2H, s, H-2). ^13^C NMR (125 MHz, CD_3_OD) δ 157.9, 156.9, 154.3, 128.4, 128.0, 127.2, 125.4, 117.1, 115.6, 115.2, 108.2, 102.1, 66.8. HRMS (DART-MS) *m*/*z* [M + H]^+^ calcd for C_15_H_13_O_3_ 241.0865, found 241.0874.

### 2.4. Synthesis of 5-OH-Eq

Genistein (2.00 g, 7.40 mmol) was placed in a stainless-steel high-pressure reactor equipped with a Teflon liner, and 10% Pd/C (1.00 g) and MeOH (10 mL) were added. The reactor was charged with H_2_ (80 atm), and the mixture was stirred at 74 °C for 72 h. After cooling to room temperature, the catalyst was removed by filtration through a Celite pad, and the filtrate was concentrated under reduced pressure. The entire resulting crude residue was divided into four approximately equal portions, each corresponding to one-fourth of the crude reaction mixture derived from 500 mg of the starting genistein, and each portion was applied to a separate preparative silica gel TLC plate (20 × 20 cm, layer thickness 2000 μm). The four plates were developed individually using toluene/acetone/MeOH (4:1:0.5, *v*/*v*/*v*). The product-containing band from each plate was collected, and the silica gel was extracted with EtOAc. The extracts obtained from all four plates were combined and concentrated under reduced pressure to afford 5-OH-Eq as a white powder (912 mg, 3.53 mmol, 48%). ^1^H NMR (500 MHz, DMSO-d_6_) δ 9.23 (1H, br s, OH), 9.16 (1H, OH), 8.92 (1H, OH), 7.06 (2H, d, *J* = 8.3 Hz, H-2′,6′), 6.68 (2H, d, *J* = 8.3 Hz, H-3′,5′), 5.86 (1H, s, H-8), 5.67 (1H, d, *J* = 2.5 Hz, H-6), 4.05 (1H, m, H-2), 3.80 (1H, m, H-2), 2.91 (1H, m, H-3), 2.70 (1H, m, H-4), 2.46 (1H, m, H-4). ^13^C NMR (125 MHz, CD_3_OD) δ 156.2, 156.1, 155.9, 155.8, 133.0, 128.1, 115.1, 101.5, 94.7, 94.4, 70.7, 37.8, 26.5. HRMS (DART-MS) *m*/*z* [M + H]^+^ calcd for C_15_H_15_O_4_ 259.0970, found 259.0962.

### 2.5. Synthesis of 5-OH-DEq

#### 2.5.1. 7-((tert-Butyldimethylsilyl)oxy)-3-(4-((tert-butyldimethylsilyl)oxy)phenyl)-5-hydroxy-4H-chromen-4-one (**3**)

Genistein (21.7 g, 80.3 mmol) was dissolved in DMF (100 mL), and imidazole (16.3 g, 240 mmol) and *tert*-butyldimethylsilyl chloride (TBSCl, 30.1 g, 200 mmol) were added successively. The reaction mixture was stirred at room temperature for 15 min and monitored by TLC (hexane/EtOAc = 15:1). The reaction was quenched with saturated aqueous NaHCO_3_, and the mixture was extracted with Et_2_O. The combined organic extracts were concentrated under reduced pressure, and the resulting residue was dried under vacuum and used directly in the next step without further purification ^1^H NMR (500 MHz, CDCl_3_) δ 12.82 (1H, s, 5-OH), 7.83 (1H, s, H-2), 7.38 (2H, d, *J* = 9.0 Hz, H-2′,6′), 6.89 (2H, d, *J* = 9.0 Hz, H-3′,5′), 6.34 (1H, d, *J* = 2.3 Hz, H-8), 6.29 (1H, d, *J* = 2.3 Hz, H-6), 0.99 (18H, s, 2× TBS *tert*-Bu), 0.26 (6H, s, Si(CH_3_)_2_), 0.22 (6H, s, Si(CH_3_)_2_). HRMS (DART-MS) *m*/*z* [M + H]^+^ calcd for C_27_H_39_O_5_Si_2_ 499.2336, found 499.2321.

#### 2.5.2. 7-((tert-Butyldimethylsilyl)oxy)-3-(4-((tert-butyldimethylsilyl)oxy)phenyl)-5-hydroxychroman-4-one (**4**)

The crude compound **3** (4.00 g) was dissolved in EtOAc, and 10% Pd/C (1.00 g) was added. After the reaction vessel had been purged with H_2_, the mixture was stirred under a hydrogen atmosphere at room temperature for 18 h. The reaction was monitored by TLC (hexane/EtOAc = 20:1). The catalyst was removed by filtration through a Celite pad, and the filtrate was concentrated under reduced pressure. The resulting residue was used directly in the next step without further purification. ^1^H NMR (500 MHz, CDCl_3_) δ 12.04 (1H, s, 5-OH), 7.13 (2H, d, *J* = 8.7 Hz, H-2′,6′), 6.82 (2H, d, *J* = 8.7 Hz, H-3′,5′), 6.00 (1H, d, *J* = 2.5 Hz, H-8), 5.95 (1H, d, *J* = 2.5 Hz, H-6), 4.58–4.49 (2H, m, H-2), 3.89 (1H, m, H-3), 0.97 (18H, s, 2× TBS *tert*-Bu), 0.26 (6H, s, Si(CH_3_)_2_), 0.19 (6H, s, Si(CH_3_)_2_). HRMS (DART-MS) *m*/*z* [M + H]^+^ calcd for C_27_H_41_O_5_Si_2_ 501.2493, found 501.2486.

#### 2.5.3. 7-((tert-Butyldimethylsilyl)oxy)-3-(4-((tert-butyldimethylsilyl)oxy)phenyl)chromane-4,5-diol (**5**)

The crude compound **4** (12.7 g) was dissolved in THF (200 mL), and LiBH_4_ (4.0 M in THF, 10 mL, 36 mmol) was added at −50 °C. The reaction mixture was stirred at the same temperature for 30 min and monitored by TLC (hexane/EtOAc = 20:1). The reaction was quenched with saturated aqueous NH_4_Cl, and the organic layer was washed with brine, dried over anhydrous Na_2_SO_4_, treated with a few drops of Et_3_N, filtered, and concentrated under reduced pressure. The resulting residue was dried under vacuum and used directly in the next step without further purification.

#### 2.5.4. 7-((tert-Butyldimethylsilyl)oxy)-3-(4-((tert-butyldimethylsilyl)oxy)phenyl)-2H-chromen-5-yl acetate (**6**)

The crude compound **5** obtained in the preceding step was dissolved in CH_2_Cl_2_ (400 mL), and Et_3_N (33.3 mL, 240 mmol) and Ac_2_O (6.9 mL, 72 mmol) were added. The reaction mixture was stirred at room temperature for 30 min and then heated at 50 °C for 90 min. The reaction was quenched with saturated aqueous NaHCO_3_, and the organic layer was washed with brine, dried over anhydrous Na_2_SO_4_, filtered, and concentrated under reduced pressure. The residue was purified by silica gel column chromatography (toluene/hexane = 4:1) to afford 6 as a white powder (4.96 g, 9.41 mmol, 12% overall yield from genistein). ^1^H NMR (500 MHz, CDCl_3_) δ 7.26 (2H, d, *J* = 8.0 Hz, H-2′,6′), 6.83 (2H, d, *J* = 8.0 Hz, H-3′,5′), 6.59 (1H, s, H-4), 6.27 (1H, d, *J* = 2.0 Hz, H-6), 6.20 (1H, d, *J* = 2.0 Hz, H-8), 5.08 (2H, s, H-2), 2.34 (3H, s, 5-OAc), 0.98 (9H, s, TBS *tert*-Bu), 0.96 (9H, s, TBS *tert*-Bu), 0.21 (12H, s, 2× Si(CH_3_)_2_). HRMS (DART-MS) *m*/*z* [M + H]^+^ calcd for C_29_H_43_O_5_Si_2_ 527.2649, found 527.2628.

#### 2.5.5. 7-((tert-Butyldimethylsilyl)oxy)-3-(4-((tert-butyldimethylsilyl)oxy)phenyl)-2H-chromen-5-ol (**7**)

Compound **6** (496 mg, 0.94 mmol) was dissolved in a mixture of THF (118 mL), H_2_O (15.5 mL), and MeOH (15.5 mL). The solution was cooled in an ice bath, and LiOH·H_2_O (71 mg, 1.69 mmol) was added. After stirring for 30 min, the reaction was quenched with saturated aqueous NH_4_Cl. The organic layer was washed with brine, dried over anhydrous Na_2_SO_4_, filtered, and concentrated under reduced pressure. The residue was purified by silica gel column chromatography (toluene) to afford 7 as a white powder (114 mg, 0.24 mmol, 25%). ^1^H NMR (500 MHz, CDCl_3_) δ 7.30 (2H, d, *J* = 8.8 Hz, H-2′,6′), 7.25 (1H, s, 5-OH), 6.92 (1H, s, H-4), 6.83 (2H, d, *J* = 8.8 Hz, H-3′,5′), 6.02 (1H, d, *J* = 2.0 Hz, H-8), 5.91 (1H, d, *J* = 2.0 Hz, H-6), 5.05 (2H, s, H-2), 0.98 (9H, s, TBS *tert*-Bu), 0.96 (9H, s, TBS *tert*-Bu), 0.20 (12H, s, 2× Si(CH_3_)_2_). HRMS (DART-MS) *m*/*z* [M + H]^+^ calcd for C_27_H_41_O_4_Si_2_ 485.2543, found 485.2524.

#### 2.5.6. 3-(4-Hydroxyphenyl)-2H-chromene-5,7-diol (5-OH-DEq)

Compound **7** (216.6 mg, 0.45 mmol) was dissolved in THF (3 mL), and tetrabutylammonium fluoride (TBAF) (1.0 M in THF, 450 μL, 0.45 mmol) was added. The reaction mixture was stirred at room temperature for 15 min. The reaction mixture was washed with phosphate buffer, and the organic layer was dried over anhydrous Na_2_SO_4_, filtered, and concentrated under reduced pressure. The residue was purified by preparative TLC (toluene/acetone/MeOH = 4:1:0.5) to afford 5-OH-DEq as a dark green powder (48.3 mg, 0.189 mmol, 42%). ^1^H NMR (500 MHz, DMSO-d_6_) δ 9.52 (2H, br s, OH), 9.36 (1H, br s, OH), 7.25 (2H, d, *J* = 8.3 Hz, H-2′,6′), 6.82 (1H, s, H-4), 6.73 (2H, d, *J* = 8.3 Hz, H-3′,5′), 5.88 (1H, d, *J* = 1.8 Hz, H-8), 5.72 (1H, d, *J* = 1.8 Hz, H-6), 4.88 (2H, s, H-2). ^13^C NMR (125 MHz, CD_3_OD) δ 158.1, 156.6, 155.1, 154.2, 129.1, 125.6, 125.3, 115.1, 112.5, 104.3, 95.6, 94.3, 66.6. HRMS (DART-MS) *m*/*z* [M + H]^+^ calcd for C_15_H_13_O_4_ 257.0814, found 257.0826.

### 2.6. Antioxidant Activity Measurements

Galvinoxyl radical (GO•) was purchased from Tokyo Chemical Industry Co., Ltd. (Tokyo, Japan) and used as received without further purification. Acetonitrile (MeCN) was purchased from Nacalai Tesque, Inc. (Kyoto, Japan) and used without additional purification. The kinetics of the reaction between GO• and the test compounds were determined by the stopped-flow technique at 298 K using a UNISOKU REP-1000-02 NM spectrophotometer (UNISOKU Co., Osaka, Japan). Equal volumes of a MeCN solution of GO• and a MeCN solution of the test compound were rapidly mixed, and the decay of the characteristic absorption of GO• at 428 nm (ε = 1.4 × 10^5^ M^−1^ cm^−1^) was monitored. The pseudo-first-order rate constants (*k*_obs_) were obtained by least-squares fitting of the absorbance decay curves using the UNISOKU kinetic analysis software (UNITCOM SOLUTION-M06M-134-UHX, Unitcom Inc., Osaka, Japan). In all experiments, plots of ln(*A* − *A*∞) versus time, where *A* and *A*∞ denote the absorbance at reaction time *t* and at infinite time, respectively, were linear over at least three half-lives with correlation coefficients (*r*) greater than 0.999. The *k*_obs_ values obtained from at least three independent measurements agreed within an experimental error of ±5%. Unless otherwise noted, all measurements were performed in air-equilibrated solutions.

### 2.7. Spectral Titrations

UV–vis spectral titrations were carried out at 298 K using an Agilent 8453 diode-array spectrophotometer (Agilent Technologies, Santa Clara, CA, USA) equipped with a Peltier temperature controller. Aliquots of a MeCN solution of DEq or 5-OH-DEq (5.16 × 10^−4^ M or 5.74 × 10^−4^ M, respectively, total volume added: 5 μL) were added stepwise to a MeCN solution of GO• (7.20 × 10^−6^ M for DEq, 8.93 × 10^−6^ M for 5-OH-DEq). After each addition, the absorption spectrum was recorded, and the absorbance at 428 nm, corresponding to GO•, was measured. The changes in absorbance were plotted against the concentration ratio of [DEq]/[GO•] or [5-OH-DEq]/[GO•]. Unless otherwise noted, all measurements were performed using air-equilibrated solutions.

### 2.8. DNA Protective Effects Against X-Ray

The DNA protective effects of antioxidants against X-ray were examined. pBR322 plasmid DNA (Takara Bio Inc., Kusatsu, Japan) was purchased and purified by ethanol precipitation. A DNA solution containing 0.1 μg of the plasmid in 10 mM phosphate buffer (pH 7.4) was mixed with 500 μM daidzein, genistein, DEq, 5-OH-DEq, or Trolox, which is known as a water-soluble analog of vitamin E (1% (*v*/*v*) acetonitrile (MeCN)). The solution was exposed to X-ray generated by a TITAN-320 system (Shimadzu Corporation, Kyoto, Japan) at 2.41 Gy/min, and the total dose was 20 Gy. The samples exposed to the X-ray were loaded onto a 1% (*w*/*v*) agarose gel at 100 ng DNA per lane and electrophoresed at 50 V for 80 min. The gel was immersed in ethidium bromide solution for staining the DNA, and the gel images were obtained under UV light exposure. The band intensities were calculated with ImageJ software (developed by Wayne Rasband, National Institutes of Health, Bethesda, MD, USA).

## 3. Results and Discussions

### 3.1. Chemistry

To investigate the biological significance of the major intestinal bacterial metabolites of daidzein and genistein, synthetic access to these metabolites was established (Figure 2). The principal reduced metabolites, Eq and 5-OH-Eq, were synthesized from daidzein and genistein, respectively. Conversion of the isoflavone (chromone) framework to the corresponding chroman skeleton requires reduction in the conjugated α,β-unsaturated carbonyl moiety. However, this transformation is challenging because the enone system is extensively conjugated with the aromatic rings and phenolic hydroxyl groups, providing substantial thermodynamic stabilization and limiting selective reduction with conventional hydride reagents. To overcome this limitation, catalytic hydrogenation using Pd/C under high hydrogen pressure was investigated. It was hypothesized that the benzylic nature of the chromone carbonyl would facilitate sequential carbonyl reduction followed by hydrogenolytic deoxygenation over the Pd catalyst, thereby directly furnishing the chroman framework. Consistent with this hypothesis, catalytic hydrogenation of daidzein and genistein over Pd/C (50 atm H_2_, 80 °C) afforded Eq and 5-OH-Eq in good yields, providing direct access to these biologically important metabolites from their parent isoflavones.

Next, DEq, an unsaturated intestinal metabolite of daidzein, was synthesized. DEq has attracted considerable interest because of its antiproliferative activity and because its scaffold is present in phenoxodiol, an anticancer drug candidate. Previous syntheses of DEq have generally relied on multistep construction of a protected chromanone intermediate. For example, Gupta and Ray prepared a protected chromanone from substituted aromatic starting materials, followed by carbonyl reduction, acid-catalyzed dehydration, and high-temperature deprotection to obtain DEq [26]. In the present study, commercially available daidzein was used directly as the starting material. Following the reduction strategy reported by Wähälä and co-workers [27], Pd/C-catalyzed transfer hydrogenation using ammonium formate afforded chromanone 1. Subsequent reduction of the carbonyl group with LiBH_4_ gave chroman-4-ol 2. Because intermediates 1 and 2 were used directly without purification, DEq was obtained in three consecutive transformations from daidzein in a 21% overall isolated yield. Treatment of 2 with TMSOTf promoted dehydration and regenerated the C-ring double bond, affording DEq.

In contrast, the corresponding genistein metabolite, 5-OH-DEq, has been identified as a microbial metabolite but has not been chemically synthesized because of its structural complexity. Applying the synthetic sequence used for DEq afforded tetrahydrogenistein; however, direct acid-catalyzed dehydration failed to provide the desired unsaturated product. This result suggested that the additional 5-hydroxyl group of genistein significantly affected intermediate reactivity and inhibited dehydration. To address this problem, the 7- and 4′-hydroxyl groups of genistein were protected as *tert*-butyldimethylsilyl (TBS) ethers to give intermediate **3**. Catalytic hydrogenation afforded chromanone **4**, and subsequent LiBH_4_ reduction at −50 °C gave the corresponding chroman-4,5-diol 5. Treatment of **5** with acetic anhydride and triethylamine afforded the dehydrated 5-O-acetyl derivative **6**. This transformation may involve transient acetylation of the secondary alcohol followed by elimination of the resulting acetate to form the thermodynamically favored conjugated C-ring double bond. However, because the putative secondary acetate intermediate was not isolated or detected, the precise reaction mechanism remains to be established. Hydrolysis of the acetate gave intermediate **7**, and final removal of the TBS protecting groups with TBAF afforded 5-OH-DEq.

To the best of our knowledge, this is the first total synthesis of 5-OH-DEq. The established synthetic strategy enables the preparation of this previously inaccessible intestinal bacterial metabolite in sufficient quantities for comprehensive physicochemical and biological evaluation. Moreover, the route should facilitate future structure–activity relationship studies and the development of structurally related analogs to elucidate the biological significance of microbial isoflavone metabolism.

### 3.2. Radical-Scavenging Activity

Soy isoflavones are generally recognized as indirect antioxidants that exert their protective effects primarily through activation of the Nrf2/ARE signaling pathway and subsequent induction of endogenous antioxidant enzymes. In contrast, flavonoids containing a catechol moiety, such as catechin and quercetin, and hydroquinone-containing antioxidants, such as vitamin E, can efficiently scavenge reactive oxygen species (ROS) through direct hydrogen atom or electron donation. Because daidzein and genistein lack these structural features, they generally exhibit limited direct radical-scavenging activity. However, intestinal microbial metabolism introduces a C-ring double bond, converting these molecules to DEq and 5-OH-DEq, respectively, and generating a stilbene-like conjugated structure that electronically connects the phenolic hydroxyl groups on the A and B rings. This structural modification is expected to enhance resonance stabilization of the phenoxyl radical generated after hydrogen atom donation, thereby increasing direct radical-scavenging activity. To test this hypothesis, the radical-scavenging kinetics of daidzein, genistein, and their intestinal bacterial metabolites were investigated using galvinoxyl radical (GO•) as a stable radical model.

GO• exhibits a characteristic absorption band at 428 nm that decreases upon reduction by an antioxidant. Radical-scavenging activity was evaluated by stopped-flow spectrophotometry in acetonitrile, in which GO• and each compound were rapidly mixed, and the decay of the 428 nm absorption was monitored under pseudo-first-order conditions with the antioxidant present in at least tenfold excess. The observed pseudo-first-order rate constants (*k*_obs_) increased linearly with increasing concentrations of DEq and 5-OH-DEq, allowing the determination of the corresponding second-order rate constants (Figure 3). The second-order rate constants were 3.13 × 10^2^ M^−1^ s^−1^ for DEq and 1.44 × 10^3^ M^−1^ s^−1^ for 5-OH-DEq. In contrast, no measurable radical-scavenging reaction was detected for daidzein, genistein, or Eq, even at substantially higher concentrations, whereas 5-OH-Eq exhibited only negligible activity, with a second-order rate constant of 3.03 M^−1^ s^−1^.

For comparison, the water-soluble vitamin E analog Trolox, a widely used reference antioxidant, exhibited a second-order rate constant of 1.10 × 10^3^ M^−1^ s^−1^ under identical experimental conditions. Although daidzein and genistein have frequently been described as antioxidants, their biological activities have generally been attributed to indirect mechanisms involving the modulation of intracellular redox signaling and antioxidant enzyme expression rather than direct radical scavenging. Consistent with this interpretation, neither the parent isoflavones nor the reduced metabolite (Eq) exhibited appreciable direct radical scavenging activity toward GO•. Similarly, the additional hydroxyl group in 5-OH-Eq alone was insufficient to confer substantial radical-scavenging activity in the absence of C-ring unsaturation.

In contrast, introduction of the C-ring double bond dramatically enhanced the radical-scavenging activity of both DEq and 5-OH-DEq. Among the compounds examined, 5-OH-DEq exhibited the highest activity, with a second-order rate constant approximately 4.6-fold greater than that of DEq and 1.3-fold greater than that of Trolox. This enhancement is most reasonably attributed to the additional 5-hydroxyl group, which increases the electron-donating character of the conjugated aromatic system and stabilizes the phenoxyl radical through extended resonance interactions. These findings indicate that 5-OH-DEq is the most potent direct antioxidant among the intestinal metabolites of soy isoflavones and demonstrate that combining C-ring unsaturation with 5-hydroxyl substitution effectively enhances radical-scavenging activity.

### 3.3. Mechanism of Radical Scavenging

The exceptionally high radical-scavenging activities of DEq and 5-OH-DEq prompted investigation of the molecular mechanisms underlying their antioxidant activity. Phenolic antioxidants generally quench free radicals through hydrogen atom transfer (HAT) or single-electron transfer (SET), and distinguishing between these pathways is essential for understanding the molecular basis of antioxidant reactivity [35,36,37]. Therefore, the effects of mechanistic probes on reaction kinetics were examined to identify the predominant radical-scavenging pathway.

To evaluate the contribution of the SET pathway, the radical-scavenging reaction was examined in the presence of Mg^2+^ (Table 1). If electron transfer contributes significantly to the reaction, the one-electron-reduced species of GO• would be expected to undergo stabilization through coordination with Mg^2+^, thereby increasing the reaction rate. However, in the presence of Mg^2+^, the second-order rate constants for 5-OH-DEq and DEq were 1.00 × 10^3^ and 2.86 × 10^2^ M^−1^ s^−1^, respectively, compared with 1.44 × 10^3^ and 3.13 × 10^2^ M^−1^ s^−1^, respectively, in its absence. The lack of significant rate enhancement indicates that stabilization of GO• did not contribute appreciably to the reaction, suggesting that the SET pathway played only a minor role.

The contribution of HAT was subsequently evaluated by measuring the reaction kinetics in the presence of acetic acid or deuterated acetic acid (CD_3_COOD). For DEq, the second-order rate constant decreased from 2.79 × 10^2^ to 1.03 × 10^2^ M^−1^ s^−1^, whereas that of 5-OH-DEq decreased from 1.10 × 10^3^ to 3.17 × 10^2^ M^−1^ s^−1^, corresponding to 2.7- and 3.5-fold decreases, respectively. These pronounced kinetic isotope effects are consistent with cleavage of the phenolic O–H bond during the rate-determining step, as replacement of hydrogen with deuterium strengthens the O–D bond and slows hydrogen atom abstraction. Therefore, the observed isotope effects provide strong evidence that radical scavenging proceeds predominantly via the HAT mechanism. Collectively, the Mg^2+^ and kinetic isotope experiments demonstrate that HAT, rather than SET, is the predominant radical-scavenging pathway of both DEq and 5-OH-DEq. These findings establish that the remarkable antioxidant activity of DEq metabolites originates primarily from efficient hydrogen donation by phenolic hydroxyl groups rather than from electron transfer.

These findings provide molecular insight into how intestinal microbial metabolism enhances the chemical reactivity of dietary isoflavones through metabolic activation, transforming relatively weak direct antioxidants into highly reactive radical-scavenging molecules. Mechanistically, introduction of the C-ring double bond generates a stilbene-like π-conjugated system that enables extensive delocalization of the phenoxyl radical formed after hydrogen atom transfer. This resonance stabilization lowers the energy barrier for O–H bond cleavage, thereby facilitating HAT to GO. Furthermore, the additional 5-hydroxyl group in 5-OH-DEq increases the electron density of the conjugated aromatic framework and provides further resonance stabilization of the resulting phenoxyl radical. These cooperative electronic effects account for the substantially higher HAT reactivity of 5-OH-DEq than that of DEq and explain why 5-OH-DEq exhibits radical-scavenging activity exceeding that of Trolox. Taken together, these findings demonstrate that the enhanced radical-scavenging activity of the DEq metabolites originates from the favorable electronic effects of intestinal microbial C-ring dehydrogenation and is further enhanced by 5-hydroxyl substitution. The molecular mechanism elucidated here provides a chemical basis for understanding the antioxidant properties of these metabolites and supports subsequent evaluation of their radical-scavenging capacity and biological protective effects.

### 3.4. Radical-Scavenging Capacity

Having established that rapid radical-scavenging activity of DEq and 5-OH-DEq occurs predominantly through HAT, their radical-scavenging capacities, defined as the number of radical equivalents that can be quenched by a single molecule, were next determined. Reaction kinetics describe how rapidly an antioxidant reacts with radicals, whereas radical-scavenging capacity reflects the number of radical equivalents that can be quenched per molecule and is therefore an equally important parameter for evaluating antioxidant efficacy.

Radical scavenging capacity was determined by UV–visible spectrophotometric titration using GO•. Increasing amounts of DEq or 5-OH-DEq were added stepwise to a GO• solution, and the decrease in its characteristic absorption at 428 nm was monitored. The molar ratio of GO• to each antioxidant required for complete disappearance of the 428 nm absorption was used to determine the number of radical equivalents scavenged per molecule. For DEq, complete disappearance of the GO• absorption was observed after addition of 0.50 equivalents, corresponding to a DEq: GO• stoichiometric ratio of 1:2 (Figure 4a). Thus, one DEq molecule scavenged approximately two radical equivalents. In contrast, complete consumption of GO• was achieved with 0.33 equivalents of 5-OH-DEq, corresponding to a stoichiometry of approximately 1:3 (Figure 4b). These results demonstrated that one 5-OH-DEq molecule scavenged approximately three radical equivalents, indicating a substantially greater radical-scavenging capacity than DEq. Although GO• is a stable model radical widely used to evaluate the intrinsic radical-scavenging properties of phenolic antioxidants, these findings suggest that DEq, particularly 5-OH-DEq, can undergo sequential antioxidant reactions, thereby enabling a single molecule to neutralize multiple radical equivalents.

The observed stoichiometry is consistent with the structural features of the two metabolites. DEq possesses two phenolic hydroxyl groups that are conjugated through its planar stilbene-like π-system. These hydroxyl groups can sequentially donate hydrogen atoms, analogous to polyphenolic antioxidants such as catechin, which undergo oxidation to the corresponding quinonoid products while scavenging approximately two radical equivalents. In contrast, 5-OH-DEq contains an additional phenolic hydroxyl group at the 5-position of the A ring. Besides increasing the electron density of the conjugated aromatic framework and stabilizing the phenoxyl radical through resonance, the 5-hydroxyl group may also serve as an additional hydrogen atom donor during sequential radical-scavenging reactions. This interpretation is consistent with the experimentally observed increase in radical-scavenging capacity from approximately two to three radical equivalents per molecule. Further structural characterization of the oxidation products generated during radical scavenging will be necessary to determine whether the 5-hydroxyl group directly participates in hydrogen atom transfer and thereby contributes to the enhanced radical-scavenging capacity.

An important finding of this study is that the superior antioxidant performance of 5-OH-DEq results from improvements in both radical-scavenging kinetics and capacity. Kinetic analysis demonstrated that 5-OH-DEq reacts substantially faster with radicals than DEq, while spectrophotometric titration showed that it also neutralizes a greater number of radical equivalents per molecule. Thus, the introduction of the 5-hydroxyl group enhances both the rate and capacity of radical scavenging, identifying it as a critical structural determinant of antioxidant efficiency.

Because reactive oxygen species are continuously generated under physiological and pathological conditions, effective antioxidants must combine rapid radical trapping with the ability to quench multiple radical equivalents sequentially. The present results demonstrate that 5-OH-DEq fulfills both requirements, exhibiting outstanding reaction kinetics and an unusually high radical scavenging capacity. These findings indicate that optimizing radical-scavenging capacity, in addition to reaction kinetics, is an important consideration in the molecular design of highly efficient phenolic antioxidants.

### 3.5. Protection Against Radiation-Induced DNA Damage

Having established that DEq and 5-OH-DEq exhibit rapid HAT-mediated radical-scavenging activity and high radical-scavenging capacity, it was next examined whether these chemical antioxidant properties could be translated into protection of biologically relevant macromolecules. The protective effects of these compounds against radiation-induced oxidative DNA damage were evaluated using the plasmid DNA pBR322. The supercoiled (SC) form of pBR322 is highly susceptible to oxidative damage. ROS generated during irradiation induce single-strand breaks, converting SC DNA to the open circular (OC) form, whereas double-strand breaks produce a linear form [38]. Because agarose gel electrophoresis distinguishes these topological forms of DNA, this assay is widely used to evaluate oxidative DNA damage and the protective effects of antioxidants.

As expected, irradiation of pBR322 markedly decreased the SC form and increased the OC form, indicating substantial oxidative DNA damage. Addition of daidzein or genistein produced little or no change in the amount of OC DNA, indicating that these compounds did not protect against radiation-induced DNA strand breaks (Figure 5). These findings are consistent with the negligible direct radical-scavenging activity of daidzein and genistein toward GO•. In contrast, 5-OH-DEq markedly preserved the SC form and suppressed OC formation, demonstrating significant protection against radiation-induced DNA damage. A comparable protective effect was observed with trolox, a water-soluble vitamin E analog widely used as a reference antioxidant and reported to exhibit radioprotective activity [39,40,41]. Therefore, the DNA-protective activity of 5-OH-DEq was comparable to that of an established phenolic antioxidant.

Despite its appreciable radical-scavenging activity toward GO• in acetonitrile, DEq did not produce a statistically significant protective effect in the plasmid DNA assay. The DNA-protection experiments were conducted in phosphate buffer containing 1% (*v*/*v*) acetonitrile as a cosolvent. Although this condition was suitable for detecting the protective effects of Trolox and 5-OH-DEq, the limited solubility of DEq remained apparent and may have lowered its effective concentration. Alternative cosolvents such as DMSO and DMF could not be used because they suppressed X-ray-induced DNA damage under the assay conditions. Accordingly, the absence of significant protection by DEq in this assay should not be interpreted as evidence that DEq intrinsically lacks DNA-protective activity. Moreover, the present results do not permit a quantitative comparison of the intrinsic protective potencies of DEq and 5-OH-DEq. Further studies using validated formulations that maintain DEq in solution without affecting radiation-induced DNA damage will be required to evaluate its intrinsic protective activity. Nevertheless, the clear protection produced by both Trolox and 5-OH-DEq confirms that the assay was capable of detecting antioxidant-dependent protection under the conditions employed.

Taken together, these findings provide proof-of-principle evidence that the chemical antioxidant properties of 5-OH-DEq can protect an isolated biological macromolecule against oxidative damage under defined cell-free conditions. Because 5-OH-DEq is generated through the intestinal microbial metabolism of genistein, these findings raise the possibility that this metabolite, rather than genistein itself, contributes substantially to the antioxidant effects of dietary genistein. Although further studies in cellular and in vivo systems are required, these findings provide an experimental basis for investigating the biological significance of 5-OH-DEq as a previously unexplored antioxidant metabolite.

## 4. Conclusions

In this study, synthetic routes were established for the major intestinal bacterial metabolites of the soybean isoflavones daidzein and genistein, including Eq, DEq, 5-OH-Eq, and 5-OH-DEq. Notably, the first total synthesis of 5-OH-DEq, an unsaturated metabolite of genistein, was achieved, providing reliable access to this previously inaccessible metabolite for systematic investigation of its physicochemical properties, biological activities, and structure–activity relationships.

Comprehensive kinetic, mechanistic, and spectrophotometric analyses demonstrated that intestinal microbial dehydrogenation fundamentally altered the intrinsic antioxidant properties of soy isoflavones. Introduction of a C-ring double bond generates a stilbene-like π-conjugated framework that enables efficient stabilization of the phenoxyl radical formed during HAT, thereby conferring potent direct radical-scavenging activity that is absent in the parent isoflavones. Among the metabolites examined, 5-OH-DEq exhibited the highest antioxidant activity, scavenging radicals predominantly through HAT, with a second-order rate constant of 1.44 × 10^3^ M^−1^ s^−1^, which exceeded that of Trolox, and a high radical-scavenging capacity of approximately three radical equivalents per molecule. These findings demonstrate that the additional 5-hydroxyl group not only accelerates radical scavenging, but also increases the overall antioxidant capacity of the DEq scaffold. Importantly, 5-OH-DEq suppressed radiation-induced oxidative damage to plasmid DNA, providing proof-of-principle evidence that its radical-scavenging activity can protect an isolated biological macromolecule under defined cell-free conditions. This finding provides a basis for further investigation of whether the chemical reactivity generated by intestinal microbial metabolism contributes to the protection of biomacromolecules under cellular or in vivo conditions.

Collectively, these findings support the concept that intestinal microbial metabolism functions as a form of metabolic activation, not only by modifying the estrogen receptor-related properties of dietary isoflavones, but also by converting relatively weak direct antioxidants into highly efficient radical-scavenging molecules. Therefore, intestinal microbial metabolism may have biological significance beyond conventional pharmacokinetic transformation, and suggests that oxidative stress regulation may represent an additional functional consequence of microbial biotransformation. Notably, 5-OH-DEq exhibited antioxidant activity superior to that of both DEq and Trolox. Because intestinal microbial metabolism generates this metabolite from genistein, these findings suggest that 5-OH-DEq, rather than genistein itself, may contribute to some of the antioxidant and cytoprotective effects traditionally attributed to dietary genistein. Although this hypothesis requires validation in cellular and in vivo systems, the present study provides the first experimental foundation for exploring the biological significance of this previously inaccessible metabolite.

Beyond its biological implications, this study provides a rational molecular design strategy for developing next-generation phenolic antioxidants. The combined introduction of C-ring unsaturation and strategic hydroxyl substitution enhanced both radical-scavenging kinetics and capacity, demonstrating an effective approach for enhancing antioxidant performance. These mechanistic insights may facilitate the design of structurally optimized antioxidants inspired by naturally occurring microbial metabolites. Studies aimed at elucidating the cellular mechanisms underlying the antioxidant and cytoprotective activities of 5-OH-DEq, together with evaluating its anticancer potential and pharmacological properties in vivo. are currently in progress. These investigations may clarify the biological significance of intestinal microbial metabolism and further establish 5-OH-DEq as an important molecular scaffold for antioxidant-based therapeutic development.

## Figures and Tables

**Figure 1:**
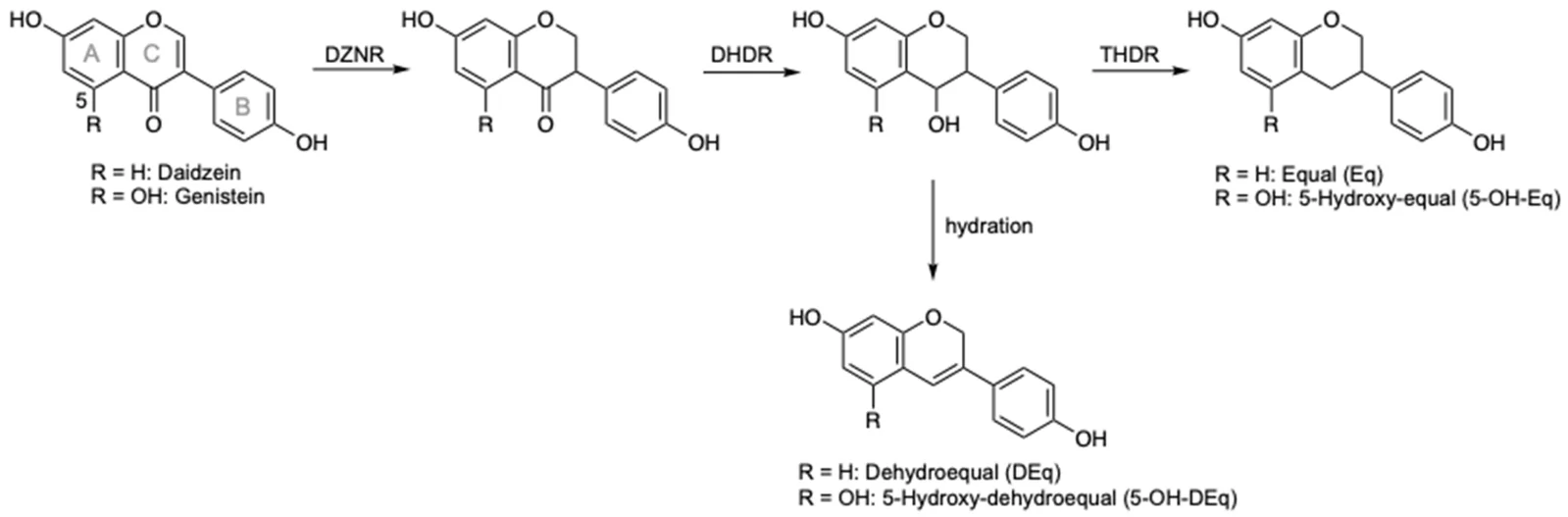
Metabolic pathways of the intestinal bacterial metabolites of daidzein and genistein. Daidzein is metabolized to equol (Eq) and dehydroequol (DEq), whereas genistein is metabolized to 5-hydroxyequol (5-OH-Eq) and 5-hydroxydehydroequol (5-OH-DEq) by intestinal bacteria. DZNR: daidzein reductase, DHDR: dihydrodaidzein reductase, THDE: tetrahydrodaidzein reductase.

**Figure 2:**
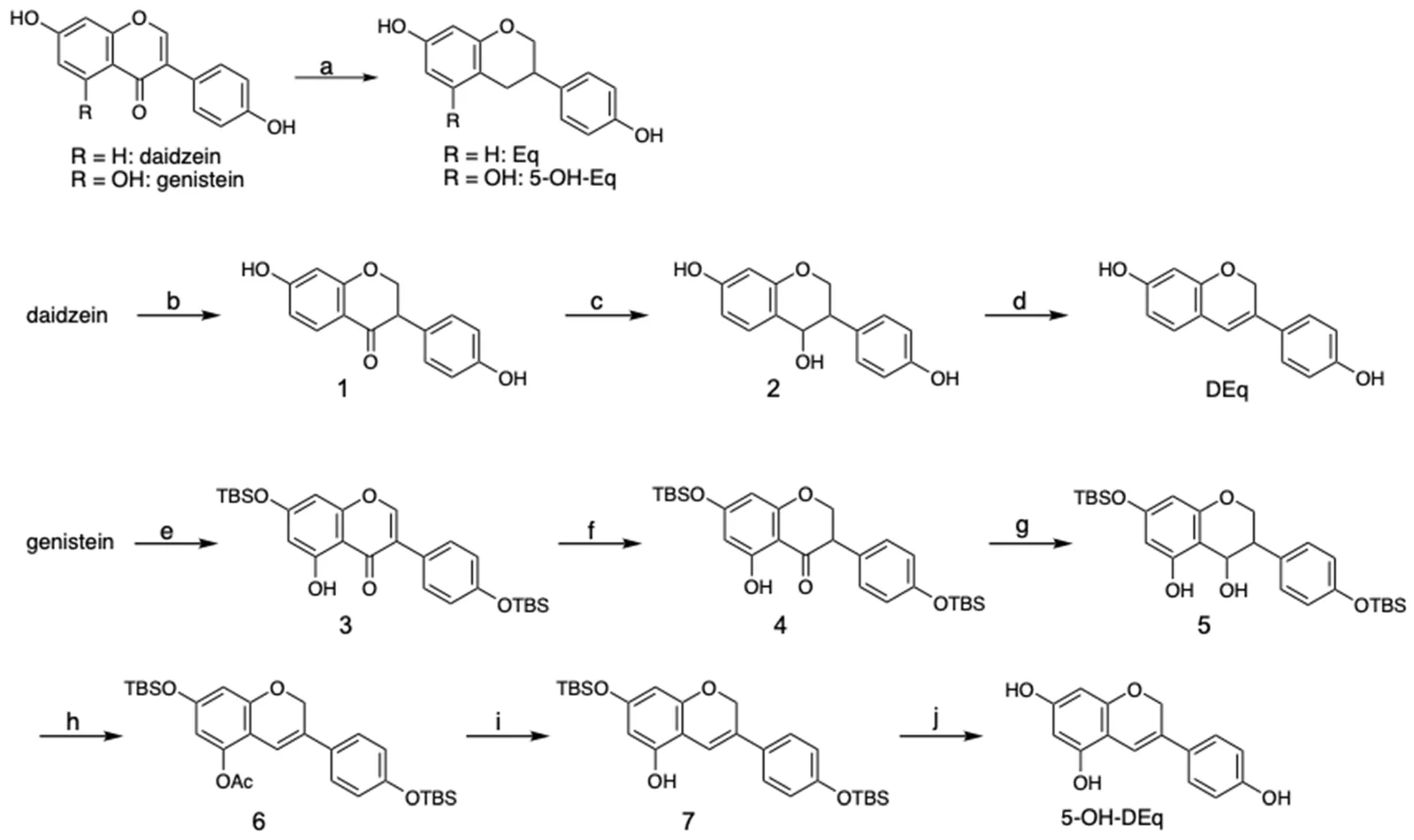
Synthetic routes to Eq, 5-OH-Eq, DEq, and 5-OH-DEq from daidzein and genistein. Eq and 5-OH-Eq were prepared by one-step catalytic hydrogenation, whereas DEq and 5-OH-DEq were synthesized through multistep procedures. Reagents and conditions: (a) 10% Pd/C, H_2_, MeOH, 50 atm, 80 °C; (b) 10% Pd/C, NH_4_OCHO, MeOH, 85 °C; (c) LiBH_4_, THF, rt; (d) TMSOTf, THF, −20 °C; (e) TBSCl, imidazole, DMF, rt; (f) 10% Pd/C, H_2_, EtOAc, rt; (g) LiBH_4_, THF, −50 °C; (h) Ac_2_O, TEA, DCM, rt; (i) LiOH, THF/MeOH/H_2_O, 0 °C; (j) TBAF, THF, rt.

**Figure 3:**
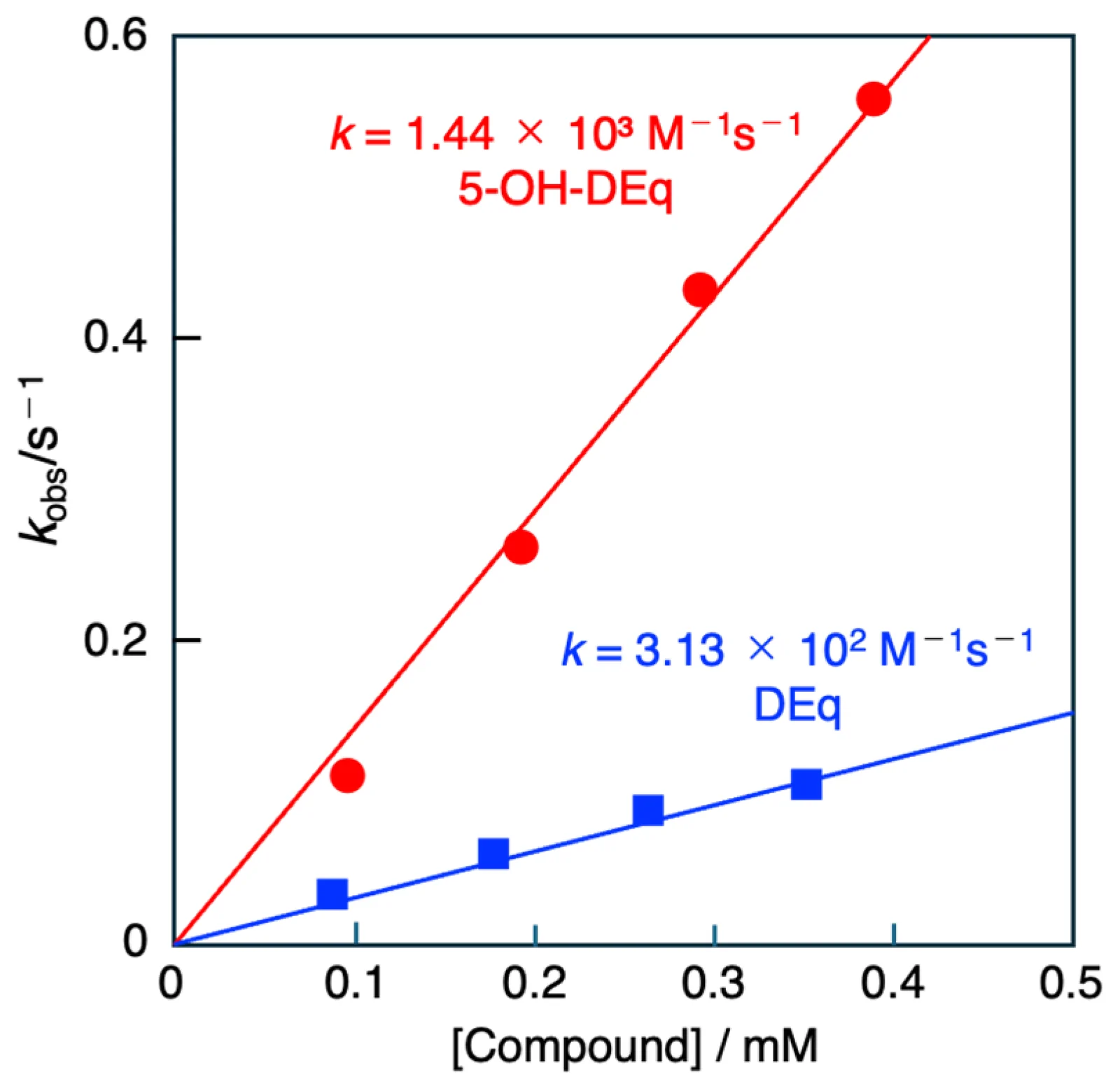
Plot of the pseudo-first-order rate constant (*k*_obs_) vs. the concentration of DEq (blue) and 5-OH-DEq (red) for the radical scavenging reaction toward GO•.

**Figure 4:**
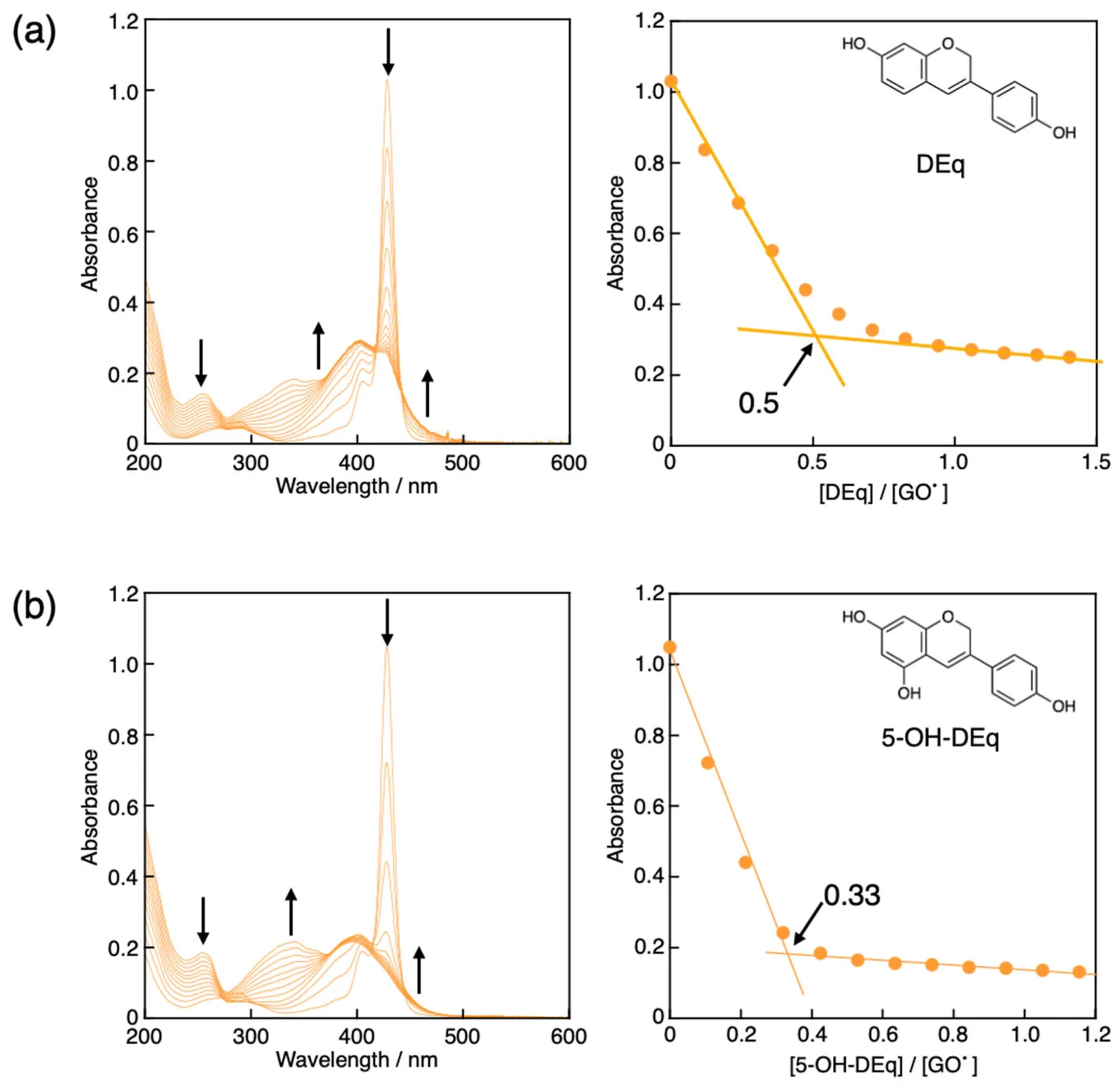
Spectral titration of GO• with DEq and 5-OH-DEq in MeCN at 298 K. (**a**) Spectral changes observed upon addition of increasing concentrations of DEq to GO• (**left**) and the corresponding plots of the absorbance at 428 nm against the concentration ratio of DEq to GO•, [DEq]/[GO•] (**right**). (**b**) Spectral changes observed upon addition of increasing concentrations of 5-OH-DEq to GO• (**left**) and the corresponding plots of the absorbance at 428 nm against the concentration ratio of 5-OH-DEq to GO•, [5-OH-DEq]/[GO•] (**right**). The arrows indicate the direction of absorbance changes.

**Figure 5:**
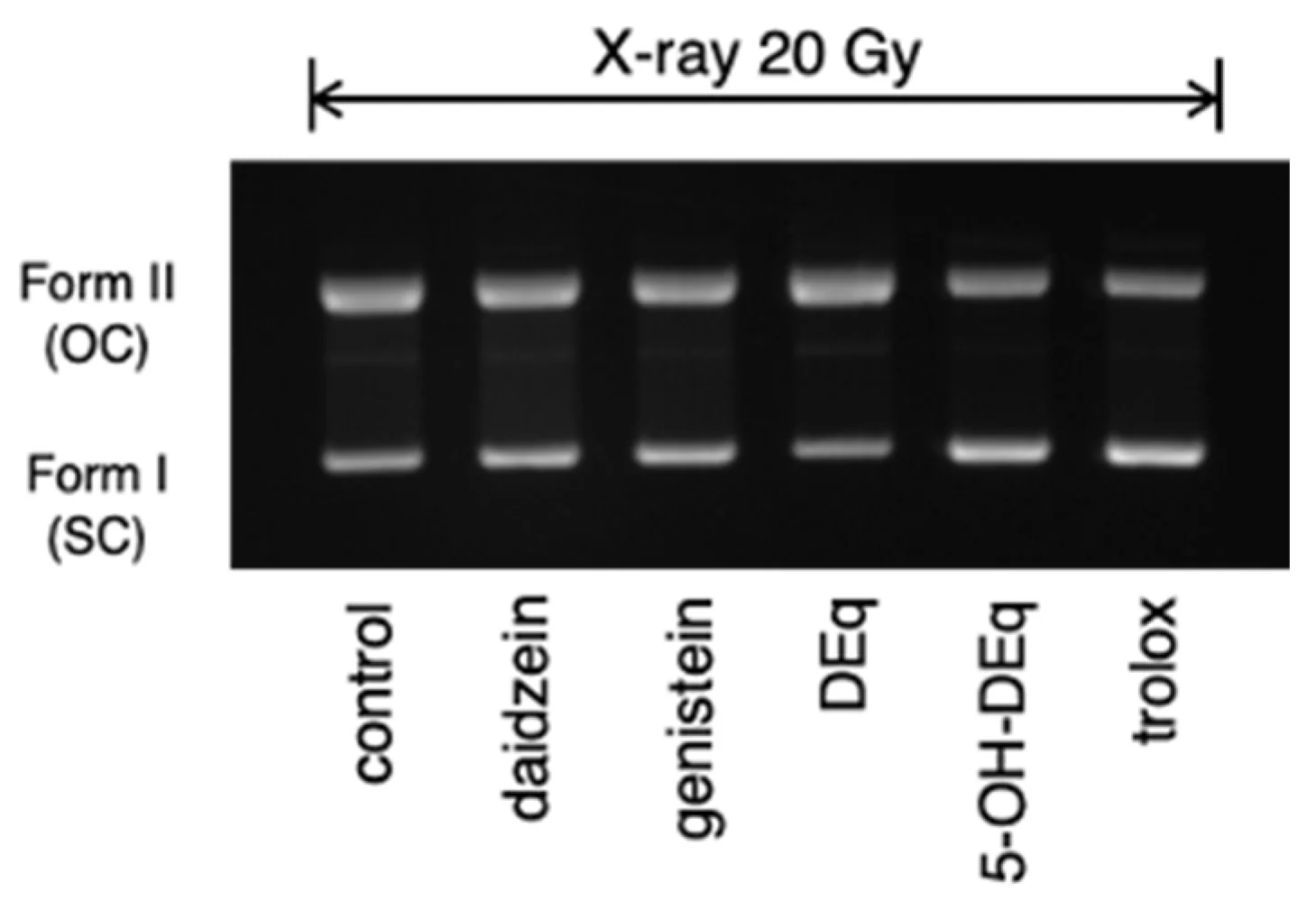
Protective effects of daidzein, genistein, DEq, 5-OH-DEq, and Trolox against X-ray-induced DNA strand breaks in pBR322 plasmid DNA. Agarose gel electrophoresis of pBR322 plasmid DNA following X-ray irradiation (20 Gy) in the absence or presence of the indicated compounds (500 µM). Irradiation converted the supercoiled (SC) form to the open circular (OC) form through radiation-induced single-strand breaks. SC, supercoiled DNA; OC, open circular DNA.

**Table 1:** Second-order rate constants for the reactions of DEq and 5-OH-DEq with GO• in the absence and presence of Mg(ClO_4_)_2_, CH_3_COOH, and CD_3_COOD. *KIE values were calculated as the ratio of the second-order rate constants measured in the presence of CH_3_COOH and CD_3_COOD (*k*_H_/*k*_D_).

Additive	DEq	5-OH-DEq
	*k*/M^−1^ s^−1^	
none	3.13 × 10^2^	1.44 × 10^3^
0.1 M Mg(ClO_4_)_2_	2.86 × 10^2^	1.00 × 10^3^
1 % CH_3_COOH	2.79 × 10^2^	1.10 × 10^3^
1 % CD_3_COOD (KIE^*^)	1.03 × 10^2^ (2.7)	3.17 × 10^2^ (3.5)

## Data Availability

All data are contained in this article.

## Supplementary Materials

The following supporting information can be downloaded at: https://www.mdpi.com/article/10.3390/antiox15101240/s1, Figure S1: ^1^H NMR and ^13^C NMR spectra of Eq, DEq, 5-OH-Eq and 5-OH-DEq.

## Abbreviations

The following abbreviations are used in this manuscript:

API	atmospheric pressure ionization
DEq	dehydroequal
Eq	equal
GO•	galvinoxyl radical
HAT	hydrogen atom transfer
HRMS	high-resolution mass spectra
MeCN	acetonitrile
ROS	reactive oxygen species
SET	single-electron transfer
TBAF	tetrabutylammonium fluoride
TBS	*tert*-butyldimethylsilyl
TBSCl	*tert*-butyldimethylsilyl chloride
TLC	thin-layer chromatography
TMS	tetramethylsilane
