# Supplementary material for: Intestinal Microbial Metabolic Activation Converts Soy Isoflavones into Potent Direct Antioxidants: First Total Synthesis and Mechanistic Characterization of 5-Hydroxydehydroequol
Source: Antioxidants (Basel). 2026 Sep 25;15(10):1240. doi: 10.3390/antiox15101240 (PMC13700811; doi:10.3390/antiox15101240)

Figure S1. ^{1}H NMR and ^{13}C NMR spectra of Eq, DEq, 5-OH-Eq and 5-OH-DEq

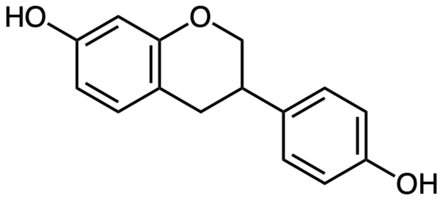
HO
O
OH

Equol (Eq)

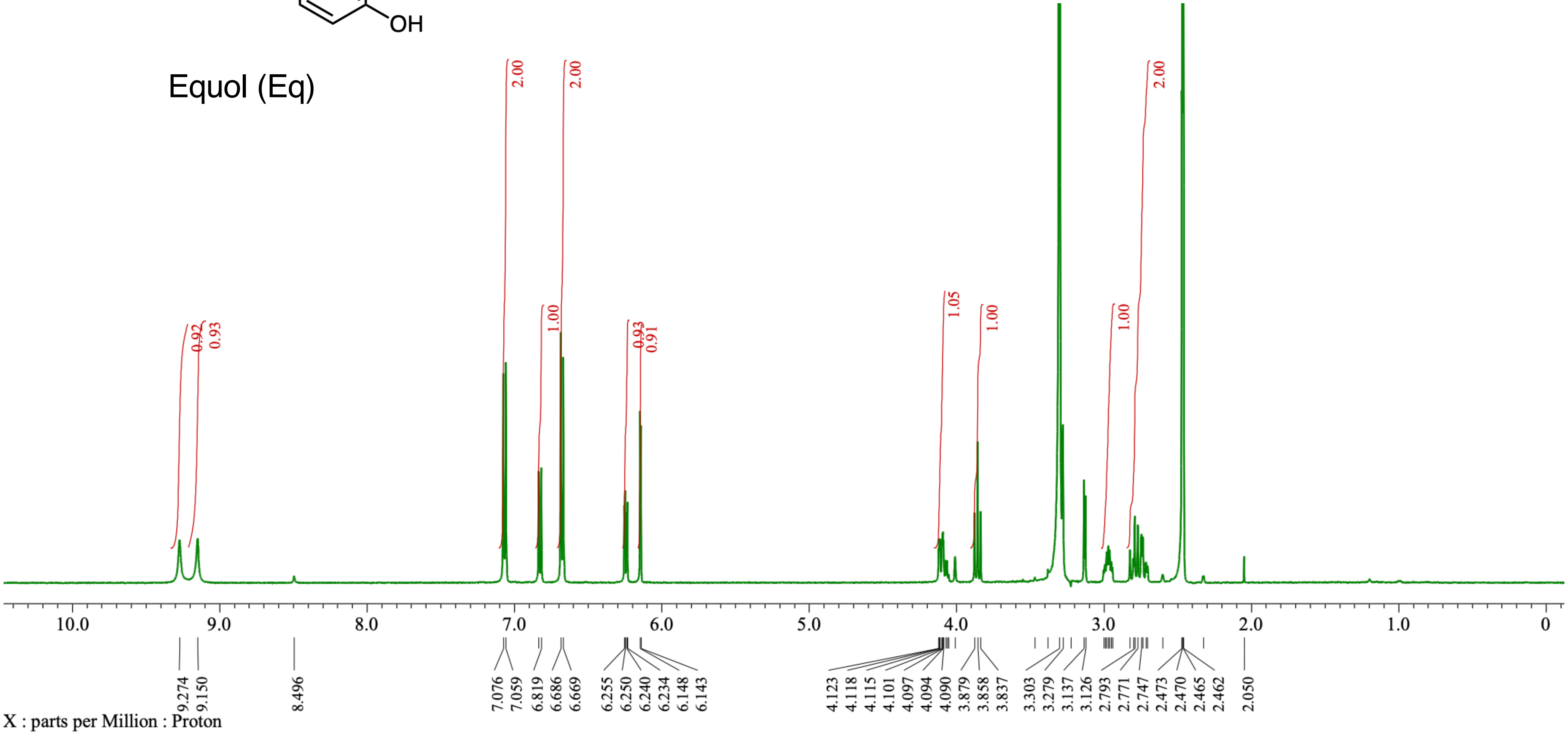
0.92
0.93
2.00
1.00
2.00
0.93
0.91
1.05
1.00
1.00
2.00
10.0
9.0
8.0
7.0
6.0
5.0
4.0
3.0
2.0
1.0
0
9.274
9.150
8.496
7.076
7.059
6.819
6.686
6.669
6.255
6.250
6.240
6.234
6.148
6.143
4.123
4.118
4.115
4.101
4.097
4.094
4.090
3.879
3.858
3.837
3.303
3.279
3.137
3.126
2.793
2.771
2.747
2.473
2.470
2.465
2.462
2.050
X : parts per Million : Proton

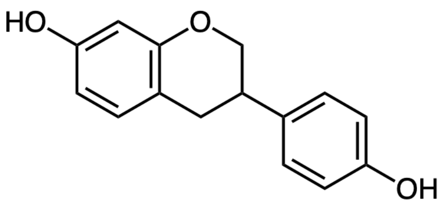
HO
O
OH

Equol (Eq)

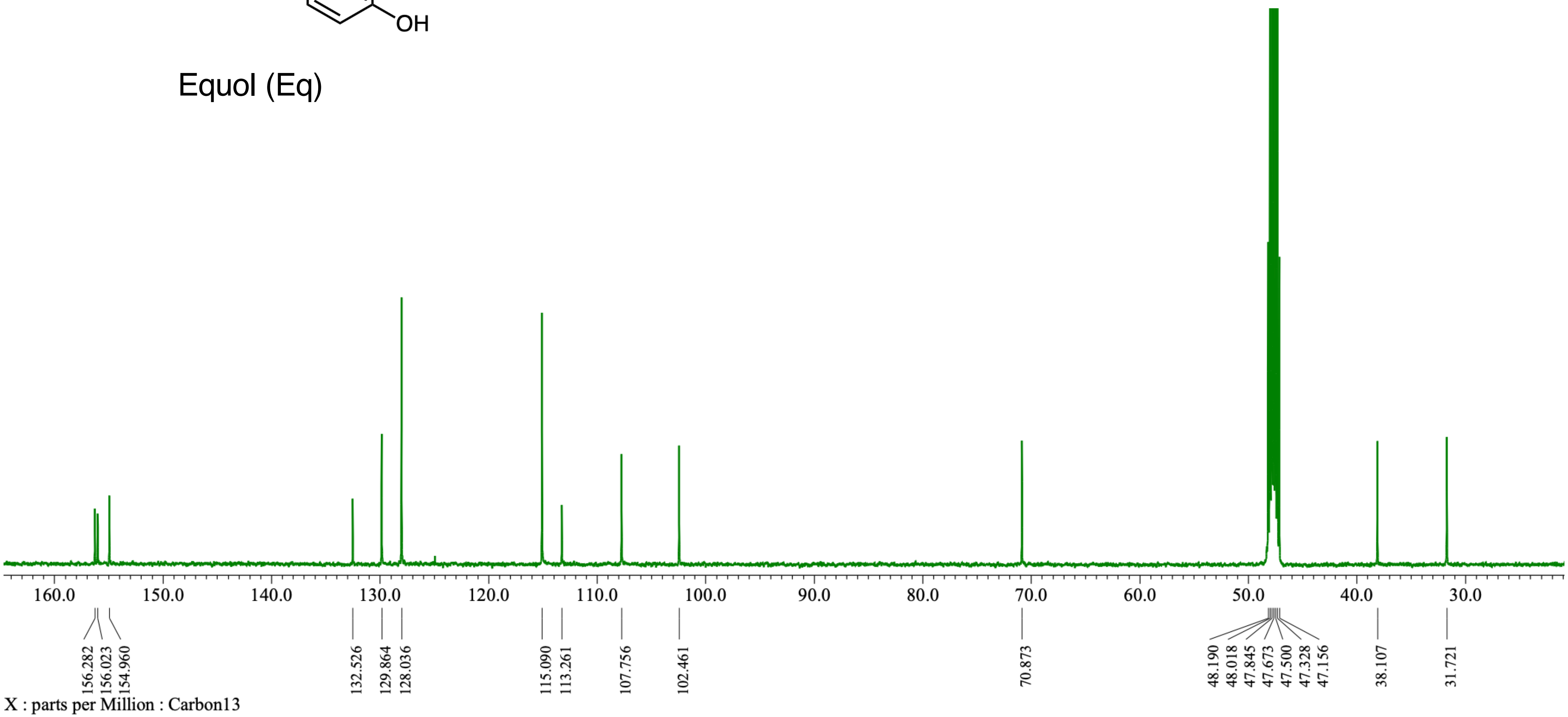
160.0
150.0
140.0
130.0
120.0
110.0
100.0
90.0
80.0
70.0
60.0
50.0
40.0
30.0
156.282
156.023
154.960
132.526
129.864
128.036
115.090
113.261
107.756
102.461
70.873
48.190
48.018
47.845
47.673
47.500
47.328
47.156
38.107
31.721
X : parts per Million : Carbon13

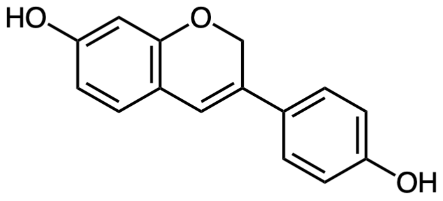

Dehydroequol (DEq)

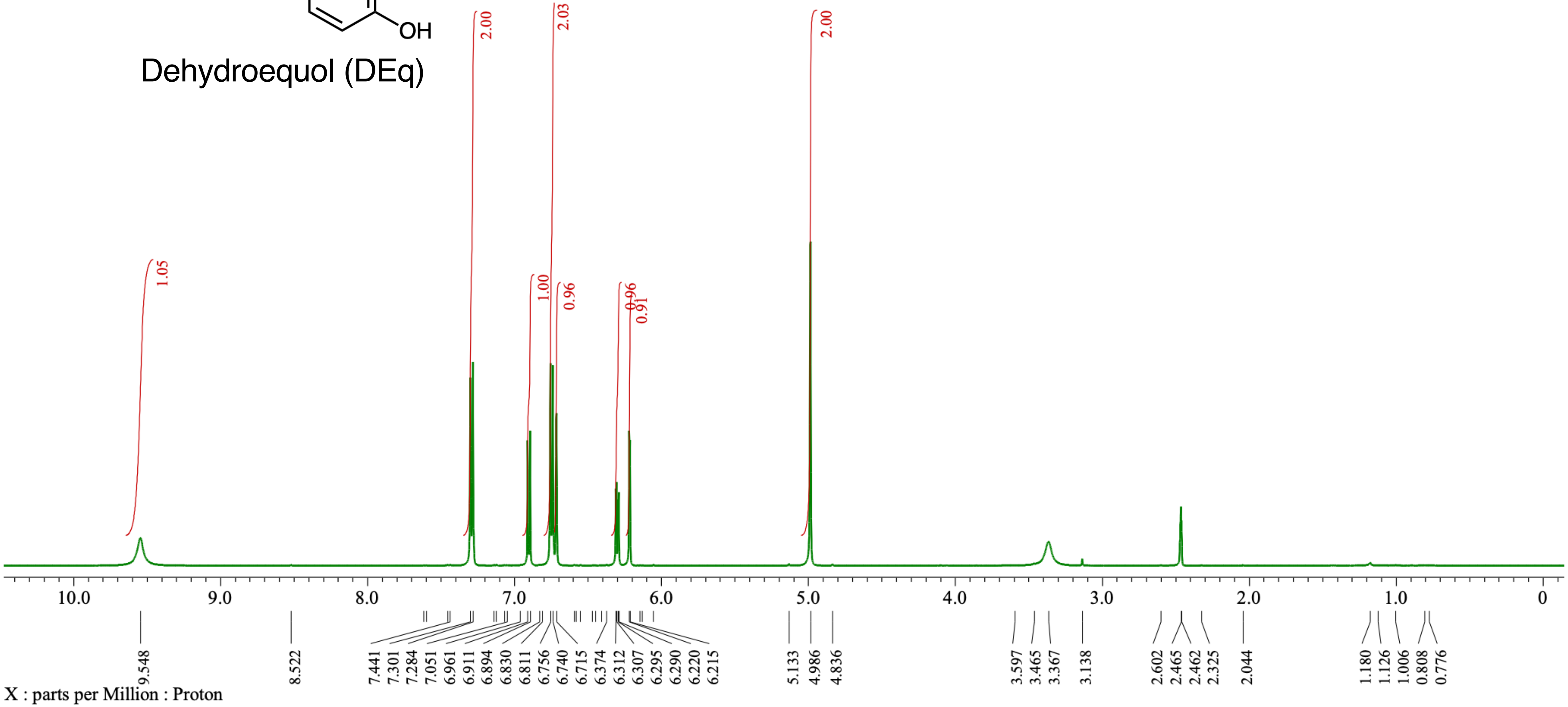

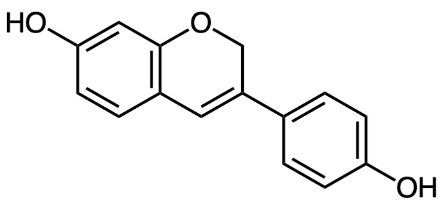

Dehydroequol (DEq)

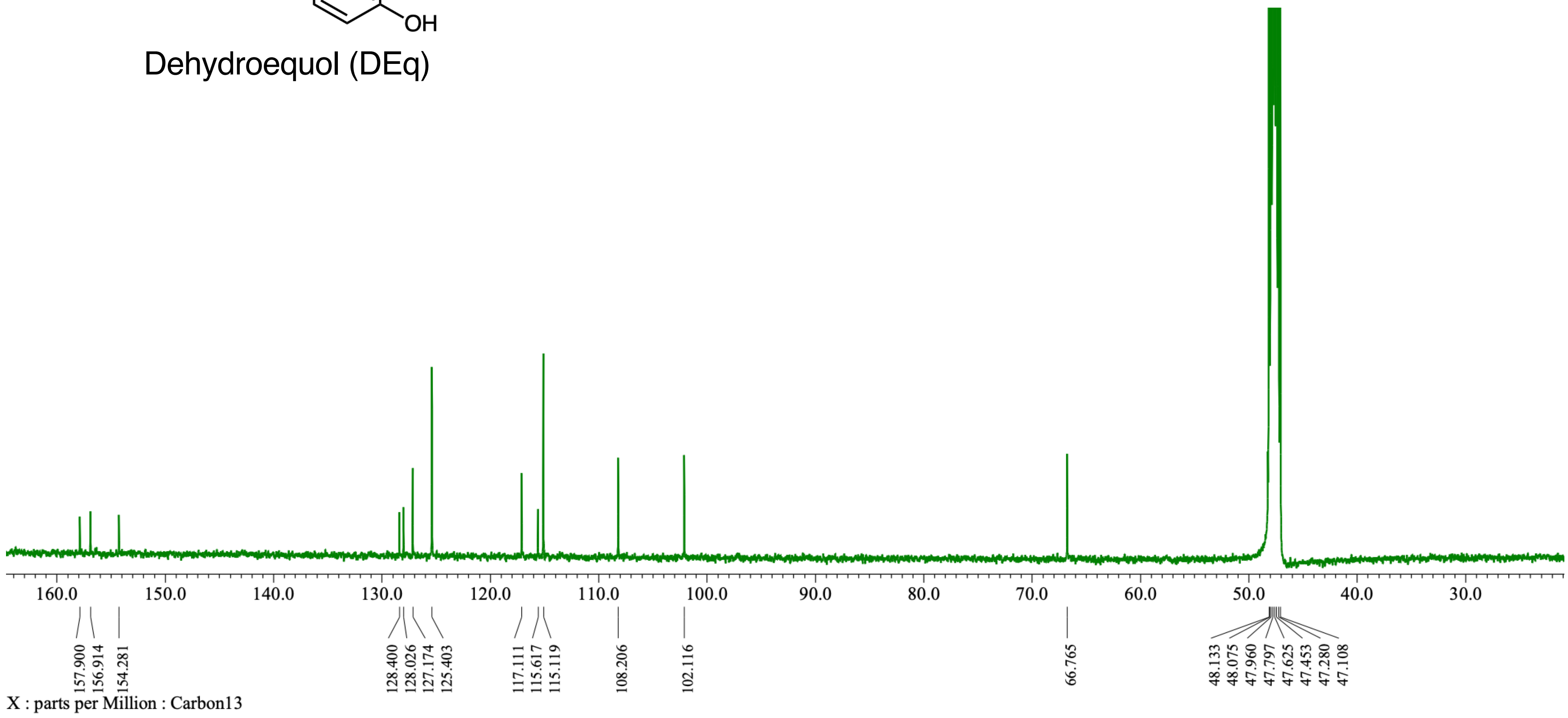

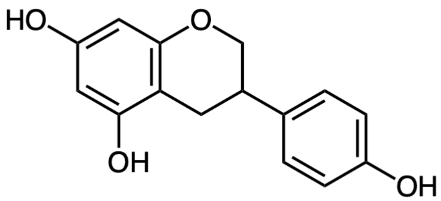

5-hydroxyequol
(5-OH-Eq)

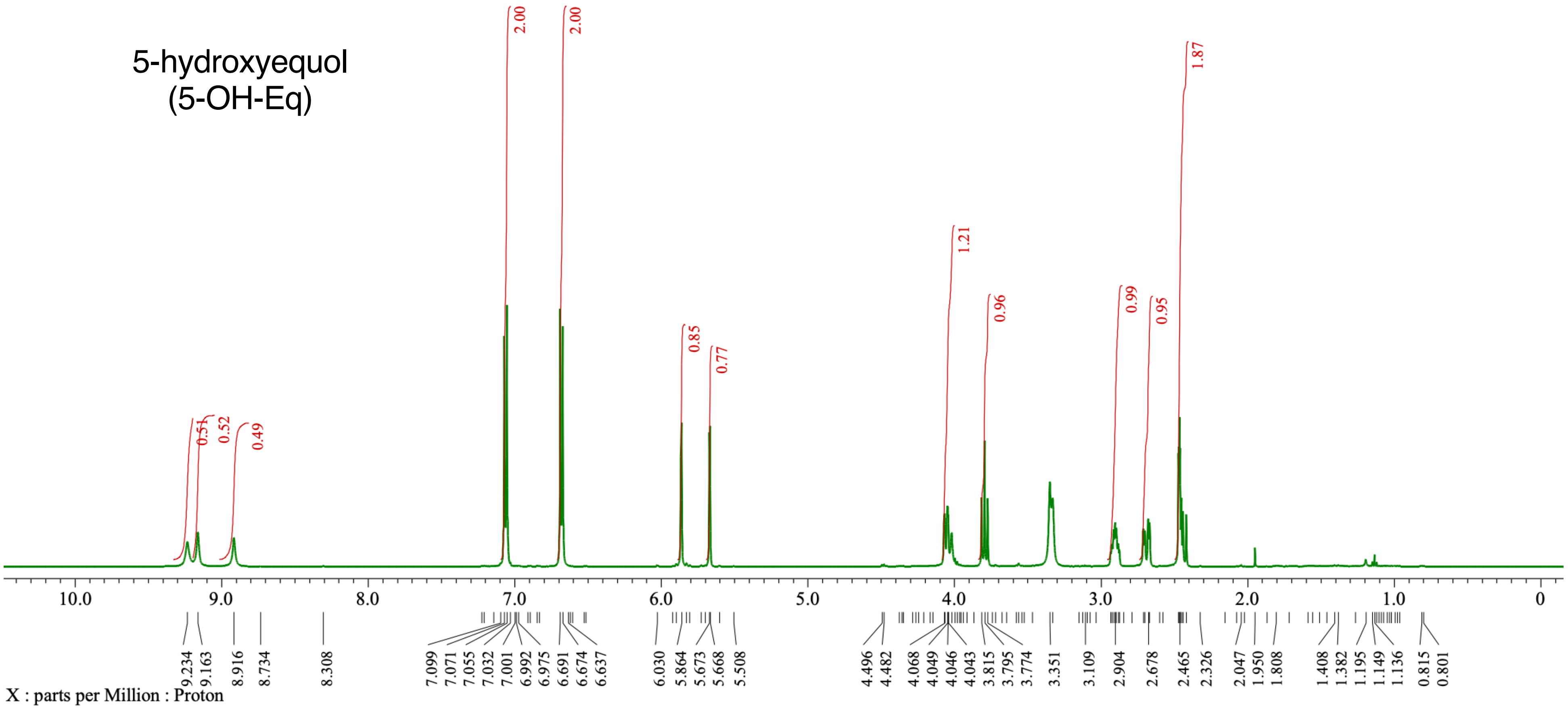

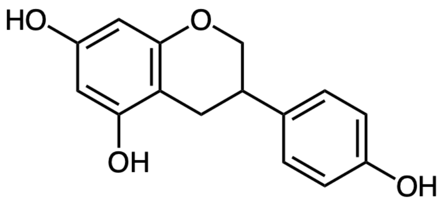

5-hydroxyequol
(5-OH-Eq)

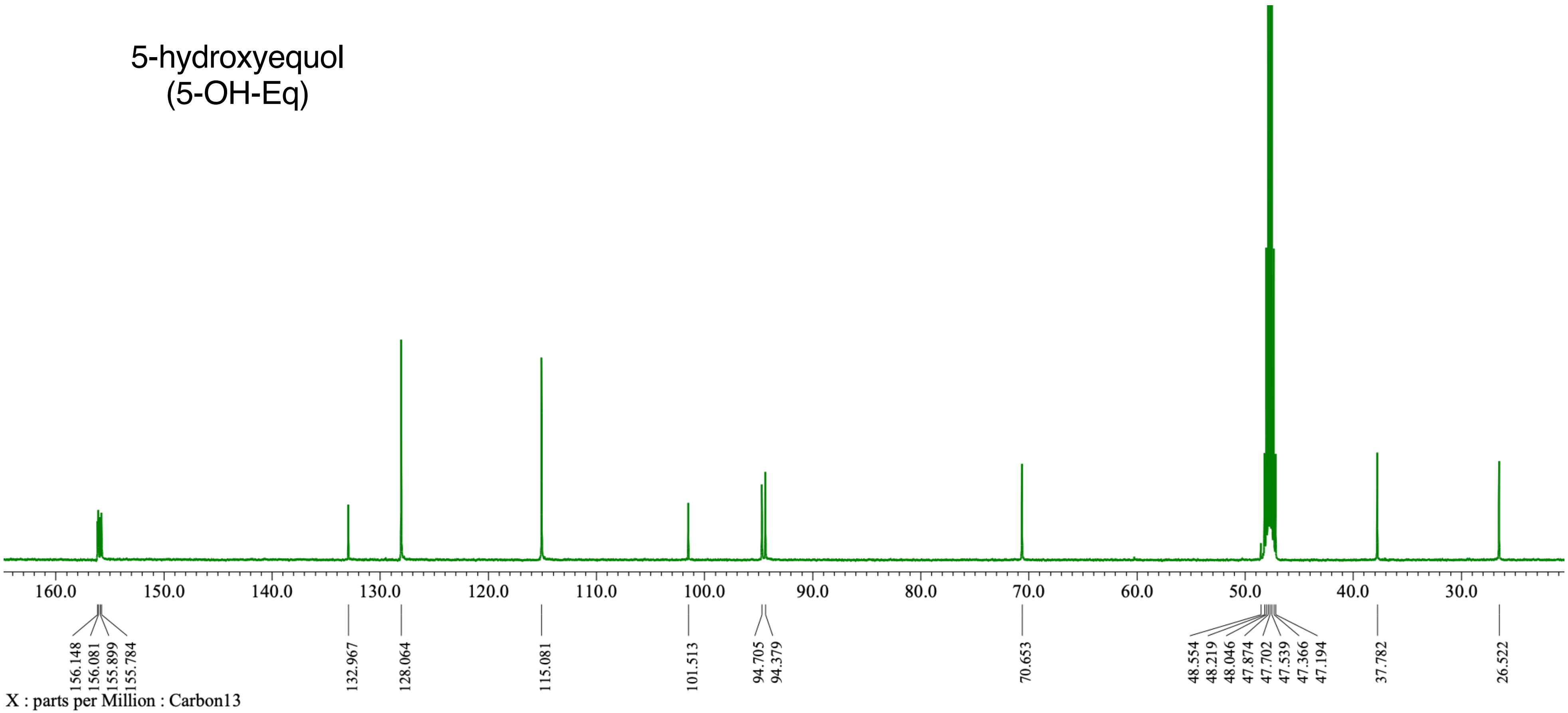

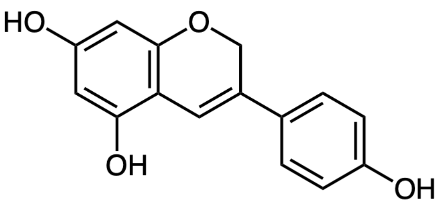

5-hydroxydehydroequol
(5-OH-DEq)

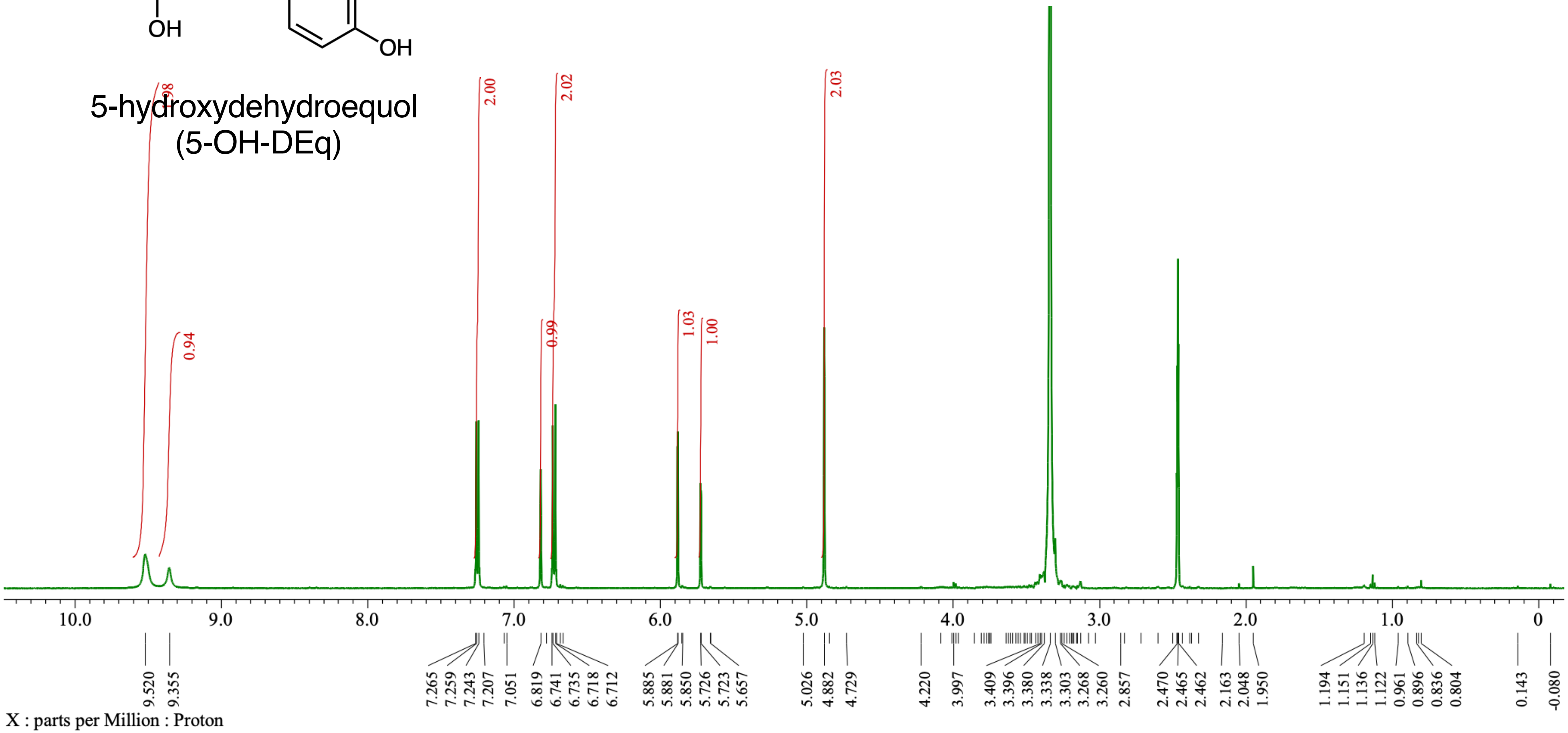

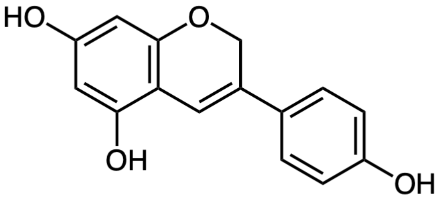

5-hydroxydehydroequol
(5-OH-DEq)

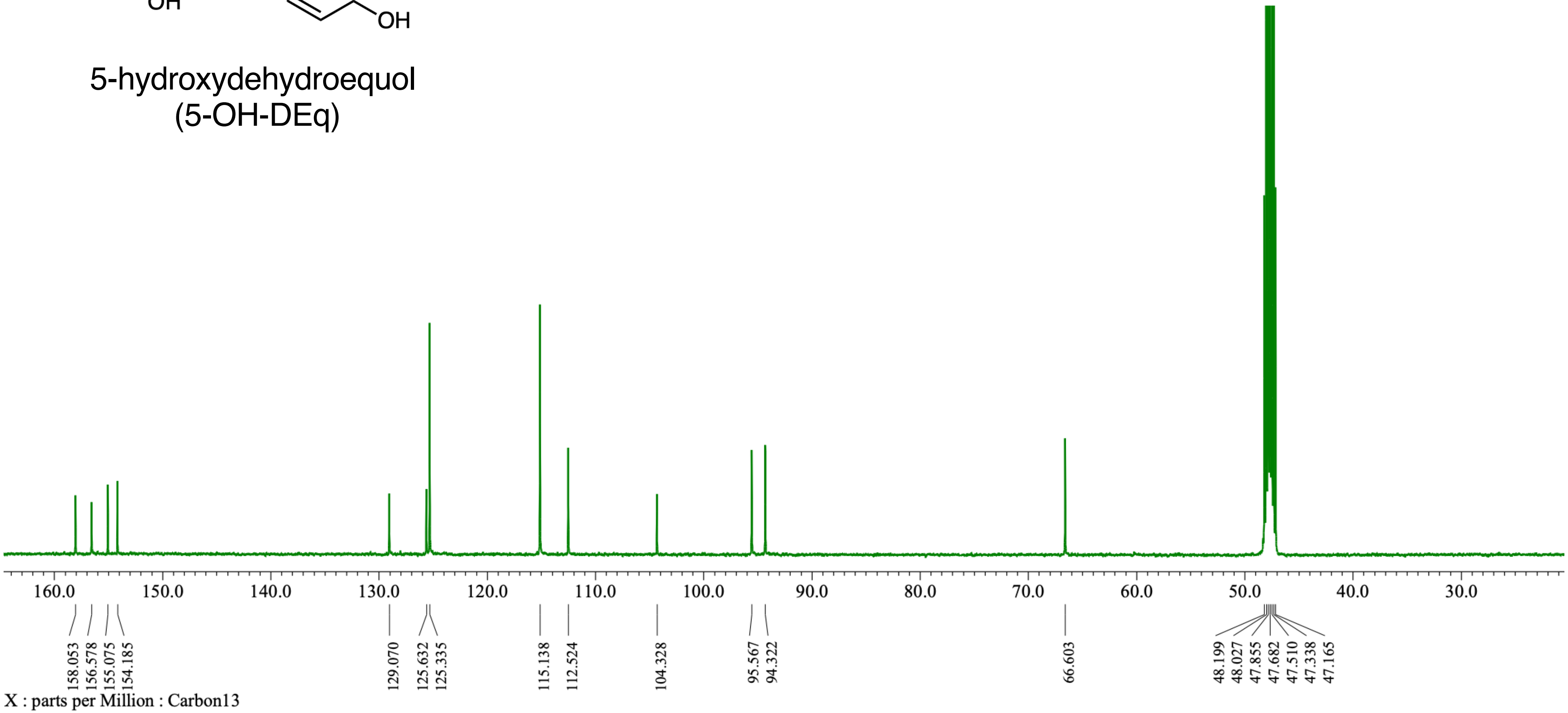

Supplement: Supplementary file 1 [file antioxidants-15-01240-s001.zip › antioxidants-4560729-supplementary.pdf]
